# Exploring the potential of the convergence between extracellular vesicles and CAR technology as a novel immunotherapy approach

**DOI:** 10.1002/jex2.70011

**Published:** 2024-09-26

**Authors:** Ofir Bar, Angel Porgador, Tomer Cooks

**Affiliations:** ^1^ The Shraga Segal Department of Microbiology, Immunology and Genetics, Faculty of Health Sciences Ben‐Gurion University Beer‐Sheva Israel

**Keywords:** chimeric antigen receptors, extracellular vesicles, immunotherapy, NK cells, T cells

## Abstract

Cancer therapy is a dynamically evolving field, witnessing the emergence of innovative approaches that offer a promising outlook for patients grappling with persistent disease. Within the realm of therapeutic exploration, chimeric antigen receptor (CAR) T cells as well as CAR NK cells, have surfaced as novel approaches, each possessing unique attributes and transformative potential. Immune cells engineered to express CARs recognizing tumour‐specific antigens, have shown remarkable promise in treating terminal cancers by combining the precision of antibody specificity with the potent cytotoxic function of T cells. However, their application in solid tumours is still in its nascent stages, presenting unique major challenges. On the same note, CAR NK cells offer a distinct immunotherapeutic approach, utilizing CARs on NK cells, providing advantages in safety, manufacturing simplicity, and a broader scope for cancer treatment. Extracellular vesicles (EVs) have emerged as promising therapeutic agents due to their ability to carry crucial biomarkers and biologically active molecules, serving as vital messengers in the intercellular communication network. In the context of cancer, the therapeutic potential of EVs lies in delivering tumour‐suppressing proteins, nucleic acid components, or targeting drugs with precision, thereby redefining the paradigm of precision medicine. The fusion of CAR technology with the capabilities of EVs has given rise to a new therapeutic frontier. CAR T EVs and CAR NK EVs, leveraging the power of EVs, have the potential to alleviate challenges associated with live‐cell therapies. EVs are suggested to reduce the side effects linked to CAR T cell therapy and hold the potential to revolutionize the penetrance in solid tumours. EVs act as carriers of pro‐apoptotic molecules and RNA components, enhancing immune responses and thereby expanding their therapeutic potential. In this review article, we navigate dynamic landscapes, with our objective being to evaluate comparative efficacy, safety profiles, manufacturing complexities, and clinical applicability.

## 1 CHARACTERISTICS, BIOGENESIS AND FUNCTION OF EVs

Extracellular vesicles (EVs) play a crucial role in intercellular communication in prokaryotes and eukaryotes (Kalluri & LeBleu, 2020) and could be broadly classified into ectosomes and exosomes (Meldolesi, 2021). While ectosomes are vesicles budding directly from the plasma membrane, exosomes arise from the endosomal pathway (Gurung et al., 2021). Exosomes typically range in diameter from 30 to 150 nm and were reported to carry a diverse range of molecular cargo, including nucleic acids, proteins, lipids, amino acids, and metabolites. As a result, EVs can serve as valuable indicators of cellular and tissue states in various diseases, hence becoming an extensively studied approach of novel theragnostic (Ebnoether & Muller, 2020), mainly due to the promising role played by EVs in cell‐to‐cell communication and their potential as diagnostic tools and therapeutic vehicles (Théry et al., 2018). Exosomes are formed through a series of regulated steps within the endocytic pathway. The process starts with plasma membrane internalization through endocytosis, followed by maturation of early endosomes into multivesicular bodies (MVBs) (Polanco et al., 2021) with intraluminal vesicles (ILVs), which eventually evolve into exosomes (Isono, 2021). The mechanism involves ESCRT and alternate pathways for ILV formation (Ju et al., 2021). ILVs within MVBs can either be degraded within lysosomes or released as exosomes (Mercier et al., 2020). The process is regulated by various molecules and proteins, including Rab GTPases, ESCRT proteins, and lipids (Jin et al., 2021). The selective packaging of specific cargo into exosomes is an active area of research (Baruah & Wary, 2020).

EVs are tiny vesicles that transport molecules to specific cells. Certain EVs contain RNA molecules that regulate gene expression and affect biological processes, particularly in disease (Correia de Sousa et al., 2019; OʼBrien et al., 2018). EVs can be loaded with cargo to have potential therapeutic applications in cancer treatment. EVs derived from cancer cells can signal cell communication and play a crucial role in stimulating cell death. This discovery offers a promising outlook for conditions such as Hepatocellular carcinoma and Glioblastoma (Ramesh et al., 2023).

### 1.1 EVs in the tumour microenvironment

#### 1.1.1 Antigen presentation

Tumour cells shed EVs, which have the capacity to be taken up by various cellular components of the tumour microenvironment (TME), including stromal, endothelial, and immune cells. Many reports indicate that by secreting EVs, tumour cells recruit the non‐cancerous cell types in the tissue and convert them to become ‘tumour‐supportive.’ Immune cells, in turn, can also shed EVs, often referred to as ‘immune system cells‐derived exosomes’ (IEXs), which can induce various responses, including transcription modulation as well as cytokine production (Hazrati et al., 2022). IEXs are thought to enhance the specificity of cytokine signals through the presence of receptors such as major histocompatibility complexes (MHCs), tetraspanins and lactadherins in the EV membrane (Benjamin‐Davalos et al., 2021). IEXs are also thought to play a role in antigen presentation (Li et al., 2023) and serve a multifaceted function in the immune system. Although they can augment anti‐tumour immunity, their impact is subject to contextual factors. In normal physiological conditions, they contribute positively to the immune response. However, in cases of conditions such as cancer, asthma, transplant rejection, and infectious diseases, their effects can be deleterious (Hazrati et al., 2022).

Notably, EVs play a vital role in adaptive immunity by presenting antigens to T cells. When shed by tumour cells EVs might carry tumour antigens which would yield various inciting immune responses. Typically, such EVs are highly enriched with programmed death‐ligand 1 (PD‐L1) and, when used as a treatment, have the capacity to suppress T‐cell activation in a dose‐dependent manner (Zeng et al., 2023). Recent studies have shown that EVs carrying MHC complexes can be efficiently internalized and processed by antigen‐presenting cells for indirect antigen presentation (Hazrati et al., 2022; Wu et al., 2021). EVs are also involved in cross‐presentation, mediating responses against viruses, tumours, vaccination, and tolerance induction (Liu & Wang, 2023; Monguió‐Tortajada et al., 2014; Santos & Almeida, 2021; Yang et al., 2021; Zonneveld et al., 2019).

A novel discovery has found that platelet‐derived extracellular vesicles (PEVs) are especially important in antigen presentation as they contain the proteasome, a crucial component needed for antigen processing and presentation to CD8+ T cells (Marcoux et al., 2021). These PEVs actively express MHC‐I and costimulatory molecules, allowing them to activate lymphocytes, increase their proliferation and cytokine production, and significantly contribute to the antigen presentation process. Further ongoing investigations are attempting to determine the role of EV surface‐associated enzymes in extracellular antigen processing (Buzas, 2023).

EVs can affect not only T cells but also natural killer (NK) cells. Resting and activated NK cells produce and secrete EVs. EVs derived from activated NK cells can activate non‐activated NK cells (Hazrati et al., 2022; Shoae‐Hassani et al., 2017). These EVs express common NK cell markers such as CD56, NKG2D, NCRs, and killer‐related proteins such as perforin and FAS‐L1 (Hazrati et al., 2022). In the context of cancer, EVs have been described as mediators of intercellular communication between cancer cells and NK cells. Tumour EVs were reported to activate NK cells, and several studies showed that NK cell EVs can modulate the immune system, rendering them a potential immunotherapeutic strategy for cancer treatment (Batista et al., 2021). Despite these novel approaches, the exact role of EVs in NK cell biology, particularly in communication with cancer cells, is still a subject of ongoing research (Wu et al., 2021).

#### 1.1.2 Potentiating the cytotoxicity of immune cells using EVs

EVs can be used to enhance the cytotoxicity of immune cells in the body. Some EV sub‐populations carrying specific surface proteins, like heat shock protein 70, can activate NK cells (Enomoto et al., 2022). This type of activation ultimately boosts the cytotoxicity of NK cells. In addition, EVs shed by NK cells were suggested to contain potent substances, including perforin, granzyme, and specific microRNAs (miR‐186 and miR‐3607) (Wu et al., 2021). These molecular cargos encapsulated inside NK‐EVs may be effective against several types of cancer cells, including breast cancer, melanoma, and neuroblastoma. Importantly, NK‐EVs may also carry typical NK cell markers such as CD56 and cytotoxicity receptors like NKG2D, enabling both tracing and their use as biomarkers (Luo et al., 2023).

On top of that, EVs derived from T lymphocytes and CAR‐T cells were also found to transport cytotoxic proteins, cytokines, and other bioactive molecules (Brizzi et al., 2022). These transported components can enhance the cytotoxic capabilities of immune cells that produce EVs, such as NK and T cells, thus strengthening their ability to target cancer cells (Calvo & Izquierdo, 2022).

#### 1.1.3 Regulatory proteins or cytokines shuttled via EVs can modulate immune dynamics

As intercellular messengers, EVs are also involved in the modulation of various immune responses. To that end, a plethora of publications indicate that regulatory proteins and cytokines, including TGF‐β, IL‐10, miRNAs, and IDO, are shuttled via EVs to recipient cells, thus modifying immune dynamics. These EV‐mediated factors down‐regulate immune recognition proteins, inducing immune tolerance in various immune cells (Tie et al., 2022). For instance, T regulatory cells (Tregs) use several immunosuppressive mechanisms, including EV secretion, to temper immune responses and target effector cells, ultimately fostering tolerance (Grover et al., 2021).

Notably, immune checkpoint molecules such as CTLA‐4, PD‐1, TIM‐3, and LAG‐3 are also considered as EV‐mediated factors inhibiting T cell activation, promoting immunosuppression and immune escape (Xing et al., 2021). They may also stimulate the differentiation of immunosuppressive cells like myeloid‐derived suppressor cells (MDSCs) and Tregs, effectively dampening the activity of immune cells such as CD8+ T cells (Joseph et al., 2022).

Dendritic cells (DCs) also secrete EVs that can be essential in immune regulation processes (Grieco et al., 2021). These EVs, known as dendritic cell‐derived EVs (DEVs), contain tumour‐associated antigens (TAAs), aid in DC maturation and facilitate specific antigen T cell activation, promoting CD4+ and CD8+ T cell responses through direct antigen presentation (Matsuzaka & Yashiro, 2022; Markov et al., 2019). DEVs exhibit heterogeneity, some of which is due to their response to various stimuli (Fernández‐Delgado et al., 2020; Kowal & Tkach, 2019). Their diversity allows for different functions and interactions with cells for physiological processes. Additionally, lymphocytes can be targeted and activated by EVs released by other immune cells, and they also release specific EVs involved in crosstalk mechanisms regulating immune responses in different contexts (Liu & Wang, 2023).

#### 1.1.4 Tumour derived EVs (TDEs)

TDEs constitute a crucial part of the intricate landscape within the TME. Shed by tumour cells, TDEs play a cardinal role in the orchestration of tumorigenesis as well as metastasis (Bai, Wang et al., 2022; Ebnoether & Muller, 2020; Fu et al., 2023; Rodrigues‐Junior et al., 2022). This multifaceted involvement extends to all hallmarks of cancer development and progression, such as angiogenesis, proliferation, and the formation of metastatic niches (Bai, Wang et al., 2022; Bao et al., 2022; Tai et al., 2019). The formation of a pre‐metastasis niche involves a complex interplay between various cells and systems, including the recruitment of bone marrow–derived cells (BMDC) (Hosseini et al., 2021). TDEs are instrumental in inducing systemic vascular leakage and recruiting macrophages to facilitate niche formation (Bai, Wang et al., 2022). They also play a role in regulating both epithelial‐mesenchymal transition (EMT), ultimately affecting tumour progression and transforming stromal cells into tumour‐associated stromal cells (TASCs) that release pro‐tumorigenic factors (Bai, Wei et al., 2022; Luo et al., 2023). Exploring TDE modulation could be a promising avenue for future chemotherapy research. As mentioned above, TDEs exhibit their capacity to impair anti‐cancer immune responses through the cargo they carry (Grieco et al., 2021). Tumour cells release EVs with the potency to suppress anti‐tumour immunity through their internalization by target cells and through receptor‐ligand interactions (Moeinzadeh et al., 2022). Packaged with a plethora of membrane‐bound proteins like Fas‐L and PD‐L1, TDEs directly hinder the activity of effector CD8+ T cells and NK cells. TDEs containing oncogenes and onco‐miRNAs have been found to bolster tumour growth through various mechanisms, including the blocking of DC maturation, induction of NK cell apoptosis, and the promotion of T cell exhaustion (Taghikhani et al., 2019). Notably, NK cells, as innate immune system effectors, usually resist tumorigenesis and metastasis while mediating tumour immune responses. However, within the TME, TDEs were observed to interfere with NK cell activity and function (Hosseini et al., 2022). Consequently, NK cell viability and cytotoxicity are compromised, leading to the evasion of immune surveillance (Huang et al., 2021).

Recent studies have shown that particular miRNAs in EVs are increased in chronic lymphocytic leukaemia (CLL) and can be used to categorize patients for personalized treatment. This emphasizes the potential of EVs as biomarkers for specific cancers and their role in precision medicine (Gargiulo et al., 2020).

The role of TDEs in remodelling the TME and pre‐metastatic niche, emphasize their potential as predictive biomarkers for tumour metastasis. Acting as central communication hubs, TDEs deliver, reprogram, and target cells, reshaping the microenvironment and contributing to pre‐metastatic niche formation. Considering these observations, it becomes evident that understanding the exosomal content and the pathways by which TDEs enable immune evasion is of paramount importance. This understanding may hold the key to revising treatment strategies and conceiving novel therapeutic approaches to surmount the obstacles in cancer treatment.

### 1.2 EVs as drug delivery vehicles

EVs have emerged as a promising avenue for advancing cancer treatment (Ma et al., 2021; Murphy et al., 2021; Sen et al., 2023). EVs offer a unique set of advantages as drug delivery systems due to their innate properties and nano‐scale size. Unlike liposomes, EVs are natural vesicles with the capability to better evade mononuclear macrophage phagocytosis, traverse extracellular matrices, and penetrate blood vessel walls, all of which facilitate efficient drug dispersion (Al Halawani et al., 2022; Sen et al., 2023). Surface molecules such as CD55 and CD59 prevent opsonin activation and coagulation factor interactions, ensuring their stable presence in biofluids (Fang et al., 2022). EVs exhibit superior biocompatibility and reduced immunogenicity compared to liposomal or other synthetic delivery systems (Lee et al., 2022). Their heterogeneity, diverse surface proteins and receptor‐mediated endocytosis facilitate effective drug internalization and steadfast bloodstream transport.

Some of the EV subsets possess homing capabilities, which make them promising carriers for a wide range of molecules, including drugs, compounds, and target nucleic acid species. Their ability to surmount biological obstacles, including the blood‐brain barrier, highlights their potential for targeted drug delivery (Dimik et al., 2023). In addition, EVs can bypass clearance mechanisms and transport therapeutic agents directly into the cytoplasm of cells, thus increasing their efficacy (Sen et al., 2023). For example, EVs carrying siRNAs have been employed in gene therapy for HER2‐positive breast cancer, and EVs showcasing a fragment of IL‐3 have exhibited encouraging outcomes in chronic myeloid leukaemia (CML) patients (Bellavia et al., 2017).

Several research groups have attempted to enhance the specificity of EVs for tumour cells by altering their surface, thereby reducing off‐target effects and ensuring precise delivery of therapeutic substances to cancer cells (Sen et al., 2023). Such targeted approaches maximize efficacy while minimizing potential side effects. For instance, researchers modified macrophage‐derived exosomes using an aminoethylanisamide‐polyethylene glycol (AA‐PEG) moiety to target lung metastases and then loaded them with paclitaxel (PTX). In‐vivo, the altered exosomes greatly enhanced the therapeutic efficacy (Chehelgerdi et al., 2023).

On the same note, the lipid bilayer membrane enveloping EVs serves as a natural protective barrier, shielding enclosed drugs from degradation and immune clearance (Chen et al., 2022; Xia et al., 2024). This innate defence not only enhances drug stability and bioavailability but also enables the delivery of a wide range of therapeutic agents (Wandrey et al., 2023). From small molecule drugs to nucleic acids such as miRNA and siRNA, and even proteins, EVs offer remarkable versatility (Murphy et al., 2021). This adaptability has paved the way for the development of combination therapies, allowing multiple drugs with complementary mechanisms of action to be co‐delivered for maximum therapeutic impact (Chen et al., 2022). Notably, EVs were proposed to simultaneously deliver chemotherapeutic drugs and microRNA inhibitors, a strategy showing promise in combating drug resistance in cancer cells (Chen et al., 2022). Additional reports showed variable methods to load EVs with therapeutic cargo. One method includes pre‐loading during EV biogenesis within the donor cell (Du et al., 2023). In a recent study (Qiu et al., 2020), MSC‐derived exosome (MSCTs‐EXO‐CTX) were loaded with a vector to deliver a combination of cabazitaxel (CTX) and tumour necrosis factor‐related apoptosis‐inducing ligand (TRAIL). The resulting particles demonstrated potent antitumor activity due to the synergistic effects of TRAIL and CTX (Du et al., 2023; Qiu et al., 2020). Other methods include post‐loading of isolated EVs, such as electroporation, sonication, incubation and the use of transfection reagents (Du et al., 2023; Ma et al., 2021; Sen et al., 2023). In another study (Li et al., 2022), EVs were reported to be loaded with doxorubicin (DOX) and lonidamine (LND) through incubation. The resulting DOX‐ and LND‐loaded EVs showed significant anticancer activity, especially the smaller EVs, which demonstrated greater inhibition of intracellular DNA synthesis, intracellular ATP inhibition, and stimulation of intracellular ROS generation (Du et al., 2023; Li et al., 2022).

EVs were also used to demonstrated the potential to transport therapeutic agents capable of reversing or bypassing resistance, thereby restoring sensitivity to treatment (Wandrey et al., 2023). Experimentally, exosomes loaded with anti‐miR‐214 have been shown to reverse chemoresistance to cisplatin in gastric cancer (Wang et al., 2018). In another study, it has been discovered that EVs from red blood cell (RBCEVs) could be used as carriers for CRISPR/Cas9 genome editing systems to target the miR‐125b‐2 locus in leukaemia cells. The study revealed that reducing the activity of miR‐125b‐2 led to an increase in BAK1, a pro‐apoptotic protein. This increased BAK1 level could potentially enhance the sensitivity of leukaemia cells to chemotherapy, thereby reducing drug resistance (Usman et al., 2018).

One of the key advantages of EVs over synthetic nanoparticles lies in their inherent biocompatibility. EVs, derived from cells, are less likely to induce adverse immune reactions compared to synthetic counterparts (Lin et al., 2024). This natural origin, combined with their small size and ability to interact with cell surface receptors, makes them readily accepted by the body and excellent carriers for therapeutic treatments (Lin et al., 2024). Moreover, the ability to engineer EVs with targeting ligands enables precise delivery of therapeutic treatments, minimizing off‐target effects and enhancing treatment specificity. Despite these promising properties, EV‐dependent therapy has major limitations on the road to becoming a robust treatment pillar. First, limited production for large‐scale applications is still a primary obstacle, notwithstanding means such as bioreactors, 3D cultures, and microfluidic devices, which have been developed to improve production (Johnston et al., 2023; Rawat et al., 2023). Additionally, these techniques might introduce diversity in the EV population, necessitating stringent purification steps (Chen et al., 2022). Physical and chemical factors can boost EV secretion; however, their impact on safety and therapeutic efficacy requires careful evaluation and standardization. The varying molecular compositions of EVs derived from the same parental cell pose a challenge in clinical application. Unknown biologically active molecules carry risks, especially in dealing with EVs secreted by tumour cells or by regenerative MSC, which can produce factors that, while promoting regeneration, might also contribute to cancerous processes. Robust characterization methods and standardized quality management practices are essential to address these issues (Liu et al., 2023).

Along the same lines, the current molecule‐loading methods for EVs are often inefficient and may compromise EVs integrity and drug stability. Innovative approaches, such as fusing exosome membrane proteins with RNA‐binding proteins, have shown promise in enhancing nucleic acid loading efficiency (Aslan et al., 2021; Jafari et al., 2020; Statello et al., 2018; Zubarev et al., 2021). Chemical reagent‐based methods that directly transfect miRNAs into EVs offer convenience and efficiency, particularly in controlling specific miRNA content (Di et al., 2023). The most meaningful factors affecting loading efficiency include the cellular source of the EV, EV‐to‐drug ratios in the loading process and drug properties. These parameters are key to any developmental stage and must be considered in formulation design (Lin et al., 2023).

Furthermore, like in other delivery vehicles, the biodistribution of EVs might become an additional hurdle. Intravenously infused exosomal drugs are rapidly cleared by the spleen and liver's reticuloendothelial system, reducing drug accumulation at target sites and potentially raising toxicity concerns (Chen et al., 2023). Strategies to prolong EVsʼ half‐life in vivo include blocking receptors involved in EV uptake and surface modification, such as enhancing CD47 expression or attaching polyethylene glycol to inhibit nonspecific uptake (Esmaeili et al., 2022; Liu & Wang, 2023). Also, miRNAs are prone to rapid degradation in vivo, posing a complex task for achieving targeted delivery (Ma et al., 2021).

While challenges persist, continuous research and development in this area are positioned to unleash the complete potential of EV‐based drug delivery, ultimately revolutionizing cancer treatment and enhancing patient outcomes. With ongoing progress in production, loading, and targeting, EV‐based therapies have the potential to transform the landscape of cancer treatment, offering renewed hope to patients and clinicians alike.

## 2 CHIMERIC ANTIGEN RECEPTORS (CARs)

### 2.1 Structure and function

Chimeric antigen receptors (CARs) are essential components of innovative immunotherapeutic approach that utilizes genetically engineered immune cells, such as T cells and NK cells, to recognize and eliminate cancer cells with high specificity. CARs are constructed with meticulous attention to three fundamental elements, each playing a crucial role in the functionality of CAR‐based therapies.

Firstly, the antigen‐binding domain, primarily derived from monoclonal antibodies, serves as the CAR's sensory component (Jayaraman et al., 2020). Structured as a single‐chain variable fragment (scFv) that is typically derived from the B cell receptor or immunoglobulin, this domain's binding affinity is of utmost importance, determining the CAR's efficiency in targeting its antigen (Xie et al., 2019). It is necessary to strike a balance, as excessively high affinity can lead to adverse effects, highlighting the need for optimal binding affinity (Caruso et al., 2015; Liu et al., 2015). CAR‐engineered cells exhibit this scFv on their surface, providing them with remarkable antigen‐specificity independently of human leukocyte antigens (HLAs) (Maus et al., 2017).

Secondly, the hinge region, strategically positioned between the antigen‐binding and transmembrane domains, offers the flexibility required for optimal contact with the target antigen. The length and composition of the hinge region influence CAR expression, signalling, and binding efficiency, with spacer length variation catering to the epitope's position on the antigen, particularly when dealing with membrane‐proximal epitopes (Zhang et al., 2023).

Thirdly, the transmembrane domain ensures the CAR's anchoring in the cell membrane, significantly impacting expression, stability, and signalling. Different transmembrane domains are selected based on the specific requirements of the CAR. Lastly, the intracellular signalling domains act as the CAR's command centre, driving cell activation and response (Min et al., 2022).

### 2.2 Generations of CARs

· First generation: These CARs have a basic structure, typically consisting of scFv and one intracellular signalling domain (usually CD3ζ). They lack co‐stimulatory domains. While they were the initial designs, they were found to have limitations in terms of immune cell activation and persistence (Zheng et al., 2023).
· Second generation: These CARs include the antigen‐binding domain and two signalling domains. They typically combine the CD3ζ signalling domain (Lee et al., 2022) with one or more co‐stimulatory domains like CD28 or 4‐1BB. This addition of co‐stimulatory domains enhances CAR T cell function and persistence (Asmamaw Dejenie et al., 2022).
· Third generation: Third‐generation CARs build upon second‐generation CARs by incorporating three signalling domains. They include the CD3ζ signalling domain and two different co‐stimulatory domains (e.g., CD28 and 4‐1BB). The goal is to provide even more robust immune cell activation and function (Grover et al., 2023).
· Fourth generation: Fourth‐generation CARs, also known as tandem CARs or TRUCKs (T cells redirected for universal cytokine killing), are still under development. They aim to have even greater control and versatility, often including inducible switches and cytokine production modules. These CARs are designed to adapt to the complex tumour microenvironment and provide more precise control over CAR behaviour (Liang et al., 2023). For example, TanCARs have been developed to target specific antigens in glioblastoma, a form of brain cancer, allowing T cells to attack a broader array of tumour cells (Guzman et al., 2023). Another advancement is the development of CD19/CD20 dual‐targeted CAR‐NK cells to intensify cytotoxicity against acute lymphoblastic leukaemia (ALL), reducing the risk of tumour escape due to antigen diversity and improving the therapy's effectiveness (Pan et al., 2020; Qin et al., 2018; Ruella et al., 2016; Wang et al., 2020).

CAR T cell therapy is a rapidly evolving field that constantly seeks to improve the structure of CARs to enhance their effectiveness against various types of cancer. The choice of CAR generation depends on the specific goals of the therapy and the unique characteristics of the targeted cancer (Bulliard et al., 2023). Recent advancements in CAR therapy, such as IL‐2 and switch receptors (Dong et al., 2021), have led to a significant decrease in side effects and an increase in overall safety (Jan et al., 2021; Zhao et al., 2021). The latest fifth‐generation CAR‐T cell therapy is now able to target BCMA and the PD‐1/PD‐L1 pathway precisely (Tomasik et al., 2022; Yuti et al., 2023), resulting in more effective cancer treatments. With the continued refinement of CAR designs, there is hope for even more efficient and safer therapies in the future (Young et al., 2022).

### 2.3 CAR T cells therapy

CAR T cell therapy is an innovative cancer treatment that genetically modifies the patient's T cells to express chimeric antigen receptors (CARs) that target specific surface antigens found on cancer cells (de Billy et al., 2022). These CAR T cells are cultured ex vivo to create a potent cancer‐fighting army that is reintroduced into the patient's bloodstream to seek out and destroy cancer cells displaying the designated target antigens (Schmidts et al., 2022). This therapy targets a wider range of antigens than traditional approaches, does not require MHC for activation, and can be directly infused into tumours, making it a highly promising cancer treatment (Dagar et al., 2023; Rafiq et al., 2020).

CAR T cell therapy is a successful treatment for relapsed or refractory acute lymphoblastic leukaemia (R/R ALL). It uses engineered CAR T cells to target the CD19 antigen and destroy cancer cells while sparing healthy ones, that had led to prolonged remissions in about 50% of patients (Aparicio‐Pérez et al., 2023). CAR (hYP7) T cells targeting GPC3 have shown great promise in treating liver cancer by inducing sustained regression of tumours (Li et al., 2020). CAR T cell therapy targeting CLDN18.2 has shown promise in eradicating gastric cancer cells while sparing stomach tissues in preclinical models (Jiang et al., 2019). A research explores HER2 CAR T cells as a potential therapy for Diffuse Intrinsic Pontine Glioma (DIPG), a highly aggressive brain tumour. The study unveils the efficacy of HER2 CAR T cells in targeting DIPG, including cases with specific mutations, and highlights the remarkable ability of these specialized CAR T cells to penetrate the brain and curtail tumour growth. The study suggests combining CAR T cell therapy with other strategies could hold the key to improving treatment efficacy (Wang et al., 2023).

CAR T cell therapy represents a groundbreaking paradigm in cancer immunotherapy. Its application spans a diverse array of cancer types, extending well beyond traditional treatment boundaries. CAR T cells offer the versatility to target a wide range of antigens, even those less prominently displayed on cancer cells. This therapy is not confined to blood‐based malignancies, as exemplified by its success in ALL (Pan et al., 2020). The therapeutic efficacy of CAR T cells is exemplified by FDA‐approved treatments targeting CD19 and BCMA, providing new hope for patients with relapsed or refractory B‐cell malignancies (Pan et al., 2022). Furthermore, CAR T cell therapy exhibits remarkable promise in the treatment of solid tumours, such as HCC and gastric cancer, with the potential for sustained tumour regression and improved outcomes. In the domain of brain tumours, CAR T cells designed to target HER2 show remarkable effectiveness against DIPG. The key takeaway is that CAR T cell therapy is an expanding frontier with vast potential, and future research may hold the key to unlocking even greater treatment efficacy, offering hope to a wide spectrum of cancer patients.

### 2.4 CAR‐induced immune cell response

Research has shown that CAR T cells can stimulate and harness the immune response against tumour cells, resulting in a better treatment outcome. These CAR T cells are particularly effective when used alongside host immune infiltrates, as they help to establish long‐lasting immunologic memory (Bulliard et al., 2023; Jin et al., 2019). Additionally, several studies have demonstrated that CAR T‐cell therapy may generate tumour‐specific T cells that capable of enhancing tumour‐killing and proliferation (Alizadeh et al., 2021). Animal experiments indicated that CAR T cells could establish immunologic memory in more established tumour microenvironments, enabling cured mice to successfully reject rechallenges with antigen‐negative tumours (Jin et al., 2019). These findings suggest that while CAR T‐cell therapy can effectively eliminate small tumours, it may not establish immunologic memory on its own.

In another recent study, the researchers have analysed the lymphoid compartment, specifically T cells, and found notable variations in gene expression following CAR T‐cell therapy in comparison to untreated mice. They also observed changes in CD4+ T cell subsets after treatment, with CD4_L1 displaying a modest increase in genes linked to effector memory CD4 T cells. Interestingly, the research revealed an unexpected finding: the interaction between two CAR components, the extracellular spacer and the cytoplasmic signalling domain, impacts the activation strength of CD8+ cytotoxic T lymphocytes (CTLs). These immune cells play a crucial role in eliminating tumour cells and releasing vital cytokines (Künkele et al., 2015).

The study investigated the effects of anti‐CD19 CAR T cell therapy on Systemic Lupus Erythematosus (SLE) patients who suffer from an autoimmune condition. After the infusion of anti‐CD19 CAR T cells, the levels of circulating serum BAFF (B cell‐activating factor) and IL‐7 increased significantly. These cytokines are crucial in regulating the immune system. The research also found that the systemic levels of inflammatory cytokines, including TNF‐α, IL‐10, and IL‐6, decreased. These changes are likely due to the depletion of B cells, which are responsible for producing these cytokines. Additionally, the study revealed that most SLE‐associated autoantibodies decreased in patients following anti‐CD19 CAR T cell therapy (Nunez et al., 2023). Also, IFNγ production resulting from CAR T‐cell activity plays a crucial role in antitumor responses. It activates T cells, reprograms macrophages/microglia cells, and enhances their ability to present antigens.

### 2.5 Challenges associated with CAR T cell therapy

#### 2.5.1 Cytokine release syndrome

Clinical manipulation of the immune arm might be accompanied by adverse side effects. One of the major challenges associated with CAR T cell therapy is the occurrence of cytokine release syndrome (CRS), characterized by the upregulation of systemic inflammatory cytokines, leading to a spectrum of clinical presentations ranging from mild symptoms such as high fever and hypotension to more severe events such as multi‐organ failure, neurotoxicity, seizures, coma and in severe cases, life‐threatening complications (Dagar et al., 2023; Rendo et al., 2022).

There are several factors that determine the incidence and severity of CRS, such as tumour burden, CAR T cell dose, CAR design, and patient characteristics. When managing this condition, it is essential to provide supportive care, closely monitor vital signs and organ function, and administer immunosuppressive agents or steroids as needed (De Philippis et al., 2023; Jain et al., 2023). To prevent or reduce the severity of CRS, researchers have developed various strategies, including modifying the CAR structure, using split or switchable CARs, incorporating safety switches or suicide genes, targeting tumour‐specific antigens, reducing tumour burden prior to infusion, and optimizing CAR T cell dose and timing (Chohan et al., 2023). While CRS is typically reversible and most symptoms resolve within 2–6 weeks, it's essential to note that mortality may occur in a small percentage of cases, usually less than 1% in most trials. Therefore, it's crucial to manage CRS effectively to minimize the risk of life‐threatening complications and optimize the benefits of CAR T cell therapy (Shaikh & Shaikh, 2023).

Although it poses challenges, CAR T cell therapy remains a powerful tool in the fight against cancer, providing hope for patients who have exhausted other treatment options. However, concerns over CRS can limit the dosage and effectiveness of this therapy. Fortunately, a recent innovative approach has emerged to address this issue involving Hu19‐CD828Z CAR T cells. These cells produce fewer cytokines, thereby reducing side effects while still delivering strong anti‐tumour effects. This breakthrough offers patients a safer treatment option without compromising effectiveness (Alabanza et al., 2017; Brudno et al., 2020).

#### 2.5.2 On‐target/off‐tumour effects

It is also worth noting that CAR‐T cell therapy may impact non‐cancerous cells alongside cancerous ones, commonly referred to as the ‘on‐target/off‐tumour effect.’ The degree of this effect is dependent on the selectivity and prevalence of the target antigen, which is frequently found in normal tissues and cells (Diep et al., 2022; Vincent et al., 2023). An example of that is the widely used CD19 antigen in CAR‐T cell therapy for B cell malignancies, which is expressed not only by cancerous B cells but also by normal B cells and some non‐B cells. Such terapeutic effort might result in B cell aplasia. A similar scenario occurs with EGFRvIII, which is used as a target antigen for CAR‐T cell therapy against gliomas. While it EGFRvIII is expressed in around 30% of gliomas, it is also found in certain normal cells in other tissues leading to potential skin toxicity and pulmonary edema in anti‐EGFRvIII CAR‐T cell therapy (Jiang et al., 2018; Ren et al., 2017).

Different approaches have been administrated to address the on‐target/off‐tumour effect. For instance, combining CAR‐T cell therapy with additional modalities or the optimization of timing and dosage of the CAR‐T cell infusion. Additionally, inhibiting immune checkpoints through CAR‐T cell therapy may be beneficial. Anti‐HLA‐G CAR‐T cell therapy can benefit even low or inconsistent HLA‐G expression (Jiang et al., 2018), while engineering CAR‐T cells to produce scFv targeting PD‐1 enhances their ability to kill tumours and improves therapeutic outcomes in animal models (Nakajima et al., 2019). For instance, engineering CAR‐T cells to express heparanase (HPSE), an enzyme that degrades heparan sulfate proteoglycans, has demonstrated enhanced extracellular matrix degradation, facilitating T cell infiltration into tumors and improving anticancer activity (Castellarin et al., 2020; Tang et al., 2015).

In essence, these studies highlight the critical need for balancing the strength of CAR signaling, carefully selecting the length of the extracellular spacer and the cytoplasmic signalling modules to ensure CAR‐T cells perform effectively and persist in the challenging tumour environment. While efforts to apply immunotherapy to solid tumours face ongoing challenges, recent studies show promise in overcoming these obstacles.

#### 2.5.3 Relapses and exhaustion

CAR T‐cell therapy has shown promise in treating various types of cancer, but relapses have remained a significant challenge. One example of this is CD19‐negative relapses, which affect up to 40% of patients. To tackle this issue, innovative strategies, such as dual antigen targeting and fourth‐generation CAR‐T cells, have been explored. However, their effectiveness in treating CD19‐negative relapses is still uncertain. When patients experience suboptimal responses or relapses, studies have shown that CART2 therapy, where the same CAR T cells are reinfused, can be effective for refractory/relapsed B‐cell malignancies (Holland et al., 2022). While CART2 therapy may not have as high remission rates as CART1, it has an excellent safety profile (Xu et al., 2023). Further research is needed to determine optimal reinfusion timing and the use of alternate CART constructs (Ji et al., 2024).

Scientists conducted research using anti‐mesothelin CAR T cells to target pancreatic cancer cells that express mesothelin. Their goal was to study the dysfunction of CAR T cells when continuously exposed to a specific antigen. The CAR T cells were repeatedly exposed to cancer cells, and an interesting discovery emerged: while they were unable to completely eliminate the cancer cells, the CAR T cells gradually lost their ability to effectively proliferate over time, indicating dysfunction (Good et al., 2021).

Recent studies are focusing on developing specific CAR T cell subsets with enhanced proliferative capacity and resistance to this exhaustion. One strategy involves creating tissue‐resident memory (TRM) CAR T cells by exposing them to TGF‐β during the ex vivo engineering process (Hou et al., 2018; Jung et al., 2023; Li et al., 2022). These CAR‐TRM cells show a stem‐like state, which helps in their persistence and strong anti‐tumour activity, despite having transcriptional features typically linked with exhaustion (Jung et al., 2023). A recent study suggests that preselecting early memory T cell subsets (TN/SCM) before manipulation could improve the effectiveness and safety of CAR T cell therapies (Singh et al., 2016). These cells have shown longer‐lasting antitumor responses, lower toxicity, and reduced severe side effects compared to standard bulk CAR T cell products (TBULK) (Arcangeli et al., 2022).

Another promising strategy involves creating CAR T cells with a stem cell memory (TSCM) phenotype (Sabatino et al., 2016; Vahidi et al., 2018). This can be achieved by co‐culturing CAR T cells with Notch ligand‐expressing feeder cells or by allowing CAR T cells to rest in the presence of IL7, IGF‐I, CXCL12, and Notch stimulation (Kondo et al., 2017). This method enhances IL2 production, proliferative capacity, and upregulates JUN expression, which prevents exhaustion and improves the cells' ability to fight tumours (Ando et al., 2021).

#### 2.5.4 In vivo versus in vitro

Interestingly, CAR constructs optimized for high activity in vitro by adjusting the extracellular spacer length or adding more signalling components in the cytoplasm were less effective at fighting tumours in vivo. Conversely, CARs with moderate signalling responses exhibited better results in eliminating tumours (Celichowski et al., 2023; Yan et al., 2023). A study revealed that overstimulating CAR T cells with repeated CAR triggering rendered these cells highly vulnerable to activation‐induced cell death (AICD) (Fajgenbaum & June, 2020; Xiao et al., 2021). Essentially, these supercharged CAR T cells were so active that they became more likely to destroy themselves (Huan et al., 2022; Lee et al., 2019).

CAR‐T cells can undergo programmed cell death, particularly when the target antigen is present and after TCR and CAR activation. This has important implications for the effectiveness of CAR‐T cell treatments for solid tumours over the long term. To address this susceptibility, various strategies are being explored, such as controlling the expression levels of CAR, targeting the CAR gene in the TCR gene locus, and developing alternative methods (Tschumi et al., 2018). A promising avenue is in vivo transduction of CAR‐T cells using Adeno‐Associated Virus (AAV) vectors, which can efficiently generate potent CAR‐T cells in vivo and promote tumour regression in humanized mouse models of T‐cell leukaemia (Qin et al., 2023; Wakao & Fukaya‐Shiba, 2023).

#### 2.5.5 Tumour heterogeneity

The inherent heterogeneity of tumours, characterized by genetic and phenotypic diversity among cancer cells within a single tumour, poses a significant challenge to the efficacy of CAR T cell therapy. This diversity can lead to the presence of cancer cell subpopulations that lack the targeted antigen, allowing them to evade CAR T cell recognition and destruction. The development of CAR T cells for solid tumours is further complicated by the limited availability of suitable target antigens (Qin et al., 2018). While common targets like CD19, BCMA, and CD20 have shown success in haematological malignancies, solid tumours require the identification and targeting of specific antigens, often with lower expression levels (Pan et al., 2020; Pont et al., 2019; Wang et al., 2020). The clinical implications of tumour heterogeneity are therefore substantial (Kailayangiri et al., 2020). In solid tumours, heterogeneity can lead to treatment resistance and relapse since CAR T cells may fail to eliminate all cancer cell populations. The presence of antigen‐negative clones can act as a reservoir for tumour recurrence, even after an initial response to therapy. The complex TME, with its immunosuppressive factors and physical barriers, further hinders the efficacy of CAR T cells, contributing to the challenges observed in clinical trials (Kailayangiri et al., 2020).

One approach to addressing the challenge of tumour heterogeneity involves targeting multiple antigens simultaneously, which reduces the likelihood of tumour escape due to antigen loss. Another strategy utilizes the IgG Fc receptor I (FcγRI) on engineered T cells, enabling them to bind to approved antibody drugs and target a broader range of tumour antigens (Tang et al., 2024). Additionally, combining CAR T cell therapy with other immunotherapies, such as oncolytic viruses and immune checkpoint inhibitors, can enhance the immune response against heterogeneous tumours (Zhou et al., 2024). The heterogeneity of target antigen expression also poses challenges in the treatment of single‐cell cancers like acute myeloid leukaemia (AML) (Saito & Nakazawa, 2024). The variability in antigen expression and the immunosuppressive TME hinder the development of effective CAR T cells. While various targets, including CD33, NKG2D, CD123, CLL‐1 and CD7, have been investigated, with some showing promise in clinical trials, especially those targeting CLL‐1 or CD123, overcoming these obstacles remains an active area of research (Saito & Nakazawa, 2024). To address tumour heterogeneity, future directions involve harnessing advanced technologies such as machine learning and multi‐omics studies (Zhou et al., 2024). These tools may have the potential to offer a more comprehensive understanding of the TME and assist in designing CARs with enhanced phenotypes, predicting patient responses, and identifying optimal combination strategies (Naghizadeh et al., 2022). Integrating these approaches holds promise in overcoming the limitations of CAR T cell therapy in solid tumours and expanding its clinical application (Achar et al., 2022).

## 3 CAR‐T EVs

The above‐mentioned limitations and side effects of CAR T therapy have pushed research efforts attempting to limit the involvement of cells. Recent years have seen an increased interest in the development of engineered EVs, specifically exosomes, for cancer treatment (Chen et al., 2022). EVs originating from CAR‐T cells have demonstrated promising abilities, including their capacity to reduce toxicity and overcome biological barriers, such as the BBB and blood‐tumour barriers (Najafi et al., 2023). Their lower toxicity potential allows them to be employed as cell‐free immunotherapy agents, potentially simplifying regulatory approval processes. Additionally, due to their low immunogenicity in heterologous infusions, CAR‐T EVs could potentially be used ‘off‐the‐shelf’ in a ‘third‐party’ setting.

CAR T‐containing Evs can penetrate solid tumours safely and efficiently, transfer RNA components that boost immune responses and restrict suppressor cells, and can deliver anticancer drugs into solid tumours for better treatment outcomes (Johnson et al., 2023). A recent study (Fu et al., 2019) showed that certain EV subsets have notable cytotoxic effects on EGFR‐ or HER2‐expressing cells. This study found CAR EVs that resist inactivation in the tumour microenvironment and suggested that the lack of PD‐1 inside these EVs play a significant role in the observed effect. The CAR EVs presented in this publication had potent antitumor effects with minimal adverse toxicity.

Proof‐of‐concept studies demonstrate the efficacy and specificity of CAR‐T EVs in killing cancer cells. They bind to and eliminate cancer cells, reaching previously inaccessible tumour sites. A novel approach integrates CD19 expression into EVs to create CAR immunotherapies. These CAR‐EVs exhibit selective cytotoxicity and demonstrate potent anti‐tumour activity with minimal toxicity, making them an effective and safe form of immunotherapy (Haque & Vaiselbuh, 2021).

Recent studies further highlight the potential of cell‐free EV therapies in terms of production and functionality (Liu et al., 2023). Autologous CAR production may benefit from CAR‐T EV therapies by avoiding the risk of reintroducing tumour cells. EVs expressing CAR can be optimized for increased release and surface expression through TCR activation and antigen stimulation strategies (Jarrige et al., 2021). Notably, EVs derived from mesothelin‐targeted CAR‐T cells show efficiency in targeting specific cancer cells with marked antitumor effects and low toxicity in vivo. Furthermore, EVs containing non‐coding RNAs, such as RN7SL1, derived from CAR‐T cells, enhance immune activation against tumour cells, demonstrating synergy with endogenous immune cells (Hort et al., 2022; Jiang et al., 2022; Ma et al., 2017)

**FIGURE 1:**
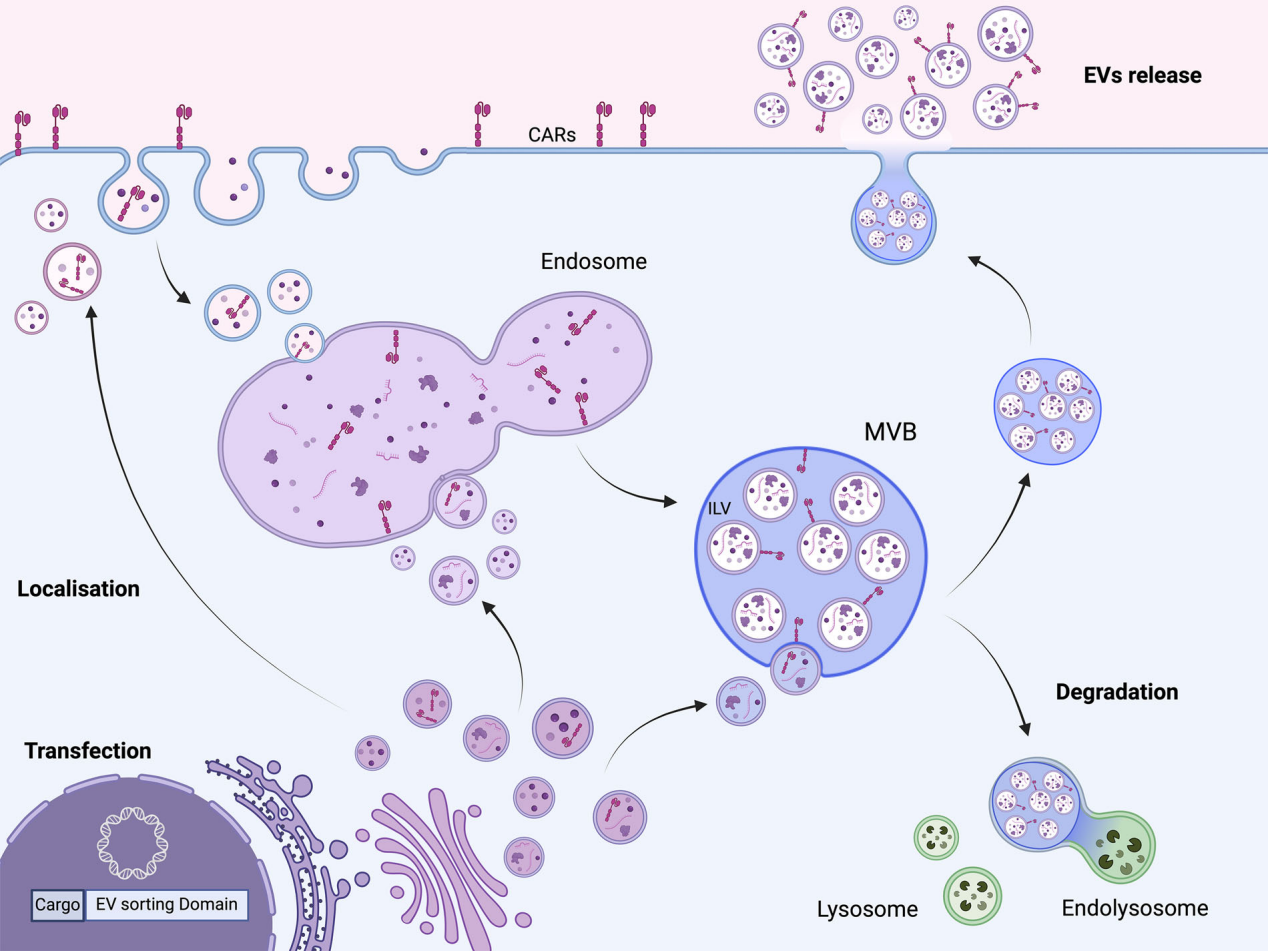
CAR on the surface or EVs released by T or NK cells. EVs are formed through a series of regulated steps within the endocytic pathway. The process starts with plasma membrane internalization through endocytosis, followed by maturation of early endosomes into multivesicular bodies (MVBs) with intraluminal vesicles (ILVs). ILVs within MVBs can either be degraded within lysosomes or released as exosomes regulated by various molecules and proteins, including Rab GTPases, ESCRT proteins, and lipids. When T cells or NK cells undergo CAR generation, the chimeric receptor localized to the plasma membrane can be invaginated and sorted into the MVBs, eventually part of the exosomal release. Alternatively, the CAR can be produced and release from the Golgi in vesicles that in turn will contain the CAR and fuse with the in any step of the endosomal process.

Ongoing research explores CAR‐T EV potential in various tumour types, compatibility with responsive and resistant tumours, and their role in more effective cancer treatments. Studies suggest that CAR EVs are well‐tolerated without toxic effects in animals tested (Zheng et al., 2023). Some studies indicate that treating mouse models with specific CAR resulted in a dose‐dependent inhibition of tumour growth. CAR EVs exhibit specific cytotoxicity against cancer cells, showing dose‐dependent tumour growth inhibition. Additionally, they offer a safer production alternative to live CAR T‐cells for solid tumour treatment. More research is required to unfold the cellular machinery sorting the CAR onto the surface of EVs. The potential mainstream manner by which the generated receptor finds its way to the EVs, can be simply by its presence on the plasma membrane and being invaginated as part of the early endosome, sorted to the MVBs and eventually shed out of the cell as a bone fide EV cargo. Nevertheless, before more studies are accumulated, we cannot exclude additional sorting mechanisms, such as CAR production through the secretory ER‐Golgi pathway to be released inside vesicles that fuse in any of the transient stages of early/late endosomal process. These options are depicted in Figure 1.

### 3.1 CAR NK cells

The emergence of CAR NK cell therapy is a highly promising development in adoptive cell therapy. It sets itself apart from CAR T cell therapies with a range of distinct advantages, including its ability to recognize target cells without relying on MHC molecules. This significantly reduces the risk of graft‐versus‐host disease and expands the potential for “off‐the‐shelf” CAR NK cell treatments (Heipertz et al., 2021; Liu et al., 2021). CAR NK cells have demonstrated exceptional safety in clinical trials focused on CD19‐positive lymphoid tumours. Their safety profile is attributed to the specific cytokine profiles displayed by activated CAR NK cells, which help limit their own toxicity (Daher & Rezvani, 2021). Additionally, their lifespan is relatively short, typically less than 10 days (Mehta & Rezvani, 2018).

The sourcing versatility and potential for allogeneic applications of CAR NK cells provide a significant advantage in their therapeutic utility. Recent research has highlighted their efficacy in targeting CD22 proteins, specifically in the treatment of oesophageal squamous cell carcinoma (Liu et al., 2023). The engineered NK cells have demonstrated robust antitumor activity, with dose‐dependent eradication of cancer cells and an increase in cytokine secretion, including significant IFN‐gamma and Granzyme B levels (Golubovskaya et al., 2023). CAR NK cell therapy represents a revolutionary immunotherapeutic strategy that combats both liquid and solid tumours. These NK cells can be sourced from patients, healthy donors, or laboratory settings, such as induced pluripotent stem cells (iPSC), which provide substantial promise for future therapeutic ventures (Khawar & Sun, 2021; Li et al., 2018).

Despite the impressive results CAR NK cell therapy has demonstrated in treating hematologic malignancies, it still encounters obstacles when applied to solid tumours. Nonetheless, the field continues to make notable progress in the engineering of NK cells, refining targets, and discovering synergistic combinations with other therapeutic agents (Burger et al., 2019).

The development of CAR‐NK cells for use in therapy poses a significant challenge. Unlike T cells and B cells, NK cells are not typically clonally expanded, making it difficult to generate, maintain, and expand CAR‐NK cells (Schmidt et al., 2021). Introducing the genetic element into the NK cell and subsequently expanding the CAR‐NK cells is a crucial step in creating NK‐CAR cells. However, the success rate of gene transduction is lower in NK cells compared to T cells (Elahi et al., 2021). Researchers have faced various obstacles, including subpar in vitro expansion and genetic modification of CAR‐NK cells, as well as a lack of optimal CAR constructs designed for NK cells. Many CAR constructs were initially developed for CAR‐T cells, and the requirement for specific extracellular surface expression of target molecules on cancer cells limits the breadth and specificity of CAR‐NK cell applications. Selecting the appropriate method for CAR transduction is critical to achieving favourable clinical results, as each method has its advantages and disadvantages.

Despite these challenges, researchers are actively exploring various methods to enhance the transduction and expansion of CAR‐NK cells. One promising approach involves developing techniques for transducing NK cells with CAR constructs directly within the patient's body, eliminating the need for ex vivo modifications. This approach may make therapy more accessible and cost‐effective. Realizing the clinical feasibility of CAR‐NK cell therapy depends on the large‐scale expansion of these cells under good manufacturing practice (GMP) standards. To address this, researchers are working to optimize freezing methods and cryoprotectants in order to preserve the cells effectively. This enables them to be offered as ‘off‐the‐shelf’ products, ensuring consistent quality for recipients (Gong et al., 2021).

A study reported that electroporation achieved high efficiency in transfecting CAR constructs into NK cells. This method allowed for the stable expression of CARs and the introduction of CAR‐NK cells with valuable innate antitumor receptors. Transient expression of CARs using electroporated plasmids is proposed as an effective and safer method, reducing the risk of insertional mutagenesis compared to viral transduction methods and being more cost‐effective.

The efficacy of CAR‐NK cell therapy is heavily influenced by the TME (Kumar & Mahato, 2023), which often creates an immunosuppressive environment that hampers the optimal functioning of CAR‐NK cells (Alzamami, 2023; Ghaffari & Rezaei, 2023). To overcome the challenges in cancer treatment, researchers are exploring several strategies. One approach is to combine CARs with checkpoint inhibitors like PD‐1. This can help block signals that impede immune cells from targeting cancer cells, which enhances the effectiveness of CAR‐NK cells (Shin et al., 2023).

Another strategy is to neutralize suppressive factors within the TME through drug interventions or engineered CAR‐NK cells that resist suppressive signals. Depleting immune suppressor cells like regulatory T cells and myeloid‐derived suppressor cells is also being investigated to increase the anti‐tumour activity of CAR‐NK cells. Designing CARs that are specific to the hypoxic TME is a promising avenue, as low oxygen levels in the TME can compromise immune cell function. Another novel approach is to use light‐responsive small‐molecule prodrugs in conjunction with CAR NK cells for drug delivery. The study also utilized 3D tumour spheroid cultures and intravital multiphoton imaging for real‐time evaluation of the therapy's effects within the tumour microenvironment (Huang et al., 2023).

Despite these strategies, there are still challenges in achieving effective NK cell therapy against solid tumours (Liu et al., 2022). Limitations such as inadequate NK cell penetration into tumours and their exhaustion within the complex TME constrain their anti‐tumour activity (Ghaedrahmati et al., 2023). Nevertheless, active research and ongoing clinical trials are dedicated to addressing these challenges, aiming to enhance the therapeutic capabilities of CAR‐NK cells (Raftery et al., 2023; Yu et al., 2024).

### 3.2 NK‐EVs

As detailed above, NK cells are key players in the innate immune system, renowned for their ability to target and eliminate virally infected and malignant cells without prior sensitization (Prager & Watzl, 2019). NK cell‐derived EVs (NK‐EVs) have recently emerged as a promising therapeutic avenue due to their inherent antitumor properties. NK‐EVs exert direct cytotoxic effects on tumour cells through the delivery of cytotoxic proteins like perforin and granzymes (Hatami et al., 2023; Kaban et al., 2021), and by transferring miRNAs that modulate gene expression in recipient cells (Brodbeck et al., 2014; Wu et al., 2019). Additionally, they induce apoptosis in cancer cells via the transfer of death receptor ligands (FasL, TRAIL, DNAM1) and can activate other immune cells, such as T cells and dendritic cells, to enhance the overall immune response against tumours. Recent studies have demonstrated the potential of NK‐EVs in combating various cancers, including acute lymphoblastic leukaemia (ALL), neuroblastoma, and breast carcinoma (Lamb et al., 2021). In vivo studies have shown their ability to accumulate specifically in tumours, cross the blood‐brain barrier, and provide sustained therapeutic benefits (Boyd‐Gibbins et al., 2022). Notably, NK‐EVs derived from non‐cancerous NK cells have exhibited anti‐tumour properties, inducing apoptosis in triple‐negative breast cancer cells without harming normal cells (Cochran & Kornbluth, 2021).

The heterogeneous nature of NK‐EVs contributes to their complex killing mechanisms. Previous research has shown that these vesicles contain varying levels of cytotoxic proteins, with perforin being a predominant component. NK‐EVs have demonstrated robust cytotoxic activity against neuroblastoma and ALL cells, with granzyme B and granulysin inducing ER stress‐mediated apoptosis (Wu et al., 2019). A nuanced understanding of their biogenesis is crucial to optimizing their clinical application in cancer therapy.

Beyond their cytotoxic effects, NK‐EVs have a suggested regulatory role in immune cell function, potentially mitigating autoimmune diseases and reducing side effects associated with immunotherapy (Cochran & Kornbluth, 2021; Matchett & Kornbluth, 2023). In vitro studies (Wu et al., 2021) have showcased their strong tumour‐targeting ability and anti‐tumour effects in hepatocellular carcinoma (HCC) mouse models, positioning them as promising candidates for immunotherapy in solid tumours (Kaban et al., 2021; Wu et al., 2021).

The use of NK‐EVs as a cancer nanomedicine is particularly appealing due to their potential to avoid severe immune responses (Cochran & Kornbluth, 2021; Matchett & Kornbluth, 2023). As nanoparticle shells originating directly from NK cells, they can be used ‘off‐the‐shelf’ in allogeneic settings, minimizing the risk of contamination with immunogenic substances. While the mechanisms underlying their tumour‐targeting abilities are not fully elucidated, their interaction with SDF‐1α suggests a potential role as prophylactic anti‐metastatic agents (Jamali et al., 2020; Wang et al., 2019). Furthermore, the combination of NK‐EVs with other drugs could overcome the challenges of cancer immunotherapy through synergistic effects (Wang et al., 2019).

### 3.3 CAR‐NK EVs

CAR‐NK cells, particularly those designed to target CD19‐positive cancers, have shown promising results in clinical trials against B‐cell malignancies, with reduced toxicities compared to CAR‐T cell therapies (Liu et al., 2020). Despite implemented strategies, challenges persist in achieving effective NK cell therapy against solid tumours, including limitations in cell penetration and exhaustion in the tumour microenvironment (Ghaedrahmati et al., 2023; Liu et al., 2022). Ongoing research and clinical trials aim to enhance CAR‐NK cell therapeutic capabilities (Raftery et al., 2023; Yu et al., 2024).

Building upon these advances, CAR‐NK‐EVs represent a novel and potentially transformative approach to cancer immunotherapy. Since NK‐EVs contain cytotoxic proteins and genetic material that mimic their parent NK cells, enabling them to induce apoptosis in tumour cells has the potential to unleash tumour cell killing (Jong et al., 2017). CAR‐NK EVs may further enhance antitumor effects by delivering cytotoxic molecules and miRNAs directly to tumour cells. It is anticipated that these vesicles, equipped with CAR proteins on their surfaces, may selectively target and bind to tumour cells expressing the corresponding antigen, facilitating the delivery of their therapeutic cargo (Hatami et al., 2023). This targeted approach not only has the potential to improve the efficacy of the treatment but also to reduces off‐target effects and toxicities.

CAR‐NK‐EVs offer several advantages over traditional cell‐based therapies. While clinical data is still emerging, the acellular nature of CAR‐NK‐EVs may translate to a more favourable safety profile compared to CAR‐T cells, potentially mitigating risks such as GVHD and CRS (Jong et al., 2017). This characteristic may contribute to a safer profile and potentially expand their applicability in a wider range of clinical settings. Additionally, CAR‐NK‐EVs could be prepared as ‘off‐the‐shelf’ products, enhancing their availability and practicality in therapeutic settings.

Recent studies have begun to shed light on the clinical potential of CAR‐NK‐EVs. Research on CD19‐positive lymphoid tumours has demonstrated that CAR‐NK cells derived from cord blood can produce potent antitumor activity (Cany et al., 2013; Liu et al., 2018). These engineered cells, which often incorporate interleukin‐15 (IL‐15) to enhance persistence and proliferation, have shown the ability to induce rapid and complete remissions in treated patients without significant adverse effects (Liu et al., 2018). Given the inherent advantages of CAR‐NK‐EVs discussed earlier (such as enhanced tumour penetration and targeted delivery), it is reasonable to hypothesize that they may also exhibit potent antitumor activity and a favourable safety profile. Further research is warranted to investigate whether CAR‐NK‐EVs, particularly those incorporating strategies like IL‐15 enhancement, can replicate or even surpass the clinical success observed with their parent CAR‐NK cells.

Both preclinical and early clinical studies have provided encouraging evidence for the efficacy of CAR‐NK‐EVs (Hatami et al., 2023). For example, Kaban et al. (2021) demonstrated that NK‐EVs loaded with BCL‐2 siRNAs effectively inhibited the overexpression of BCL‐2 in breast cancer cells, significantly enhancing tumour cell killing. Similarly, Boyd‐Gibbins et al. (2022) reported that CAR‐NKEVs carrying miR‐186 inhibited tumour growth and countered immune escape mechanisms in neuroblastoma.

The potential of CAR‐NK‐EVs may extend to various cancer models. Their ability to penetrate tumour tissues more effectively than NK cells, due to their smaller size, and their capability to be engineered for specific targeting, make them a versatile tool in cancer therapy. Additionally, CAR‐NK EVs can be stored long‐term, ensuring readiness for therapeutic use. The anti‐tumour activity of EVs has been demonstrated in preclinical studies for various cancers, including melanoma, breast cancer, and liver cancer. In these studies, EVs successfully inhibited tumour growth and improved survival rates in animal models.

Despite their significant potential and similarly to CAR‐T‐EVs, the development and clinical application of CAR‐NK‐EVs encounter several challenges. The heterogeneity of EVs, which is influenced by the origin and activation status of parent NK cells, complicates quality control and our overall understanding of their function. Standardized protocols for NK cell culture and EV isolation, specifically CAR‐NK‐EVs, are needed to ensure consistent production and therapeutic effectiveness. Furthermore, enhancing the production efficiency of NK‐EVs and CAR‐NK‐EVs is essential to lower costs and facilitate clinical translation.

Furthermore, despite promising results in preclinical studies, additional research is required to determine the optimal dosage, administration routes, and long‐term efficacy of NK‐EV therapies in humans and of CAR‐NK‐EVs in humans.

Overall, CAR‐NK EVs represent a promising new approach in cancer immunotherapy, combining the inherent advantages of NK cells with the specific targeting capabilities provided by CAR technology. Their ability to mediate direct cytotoxicity, modulate the immune environment, and deliver targeted therapies positions them as attractive candidates for further clinical development. Future studies should focus on optimizing production methods and functional properties to maximize their therapeutic potential while ensuring safety and efficacy in clinical settings.

## 4 SUMMARY

**FIGURE 2:**
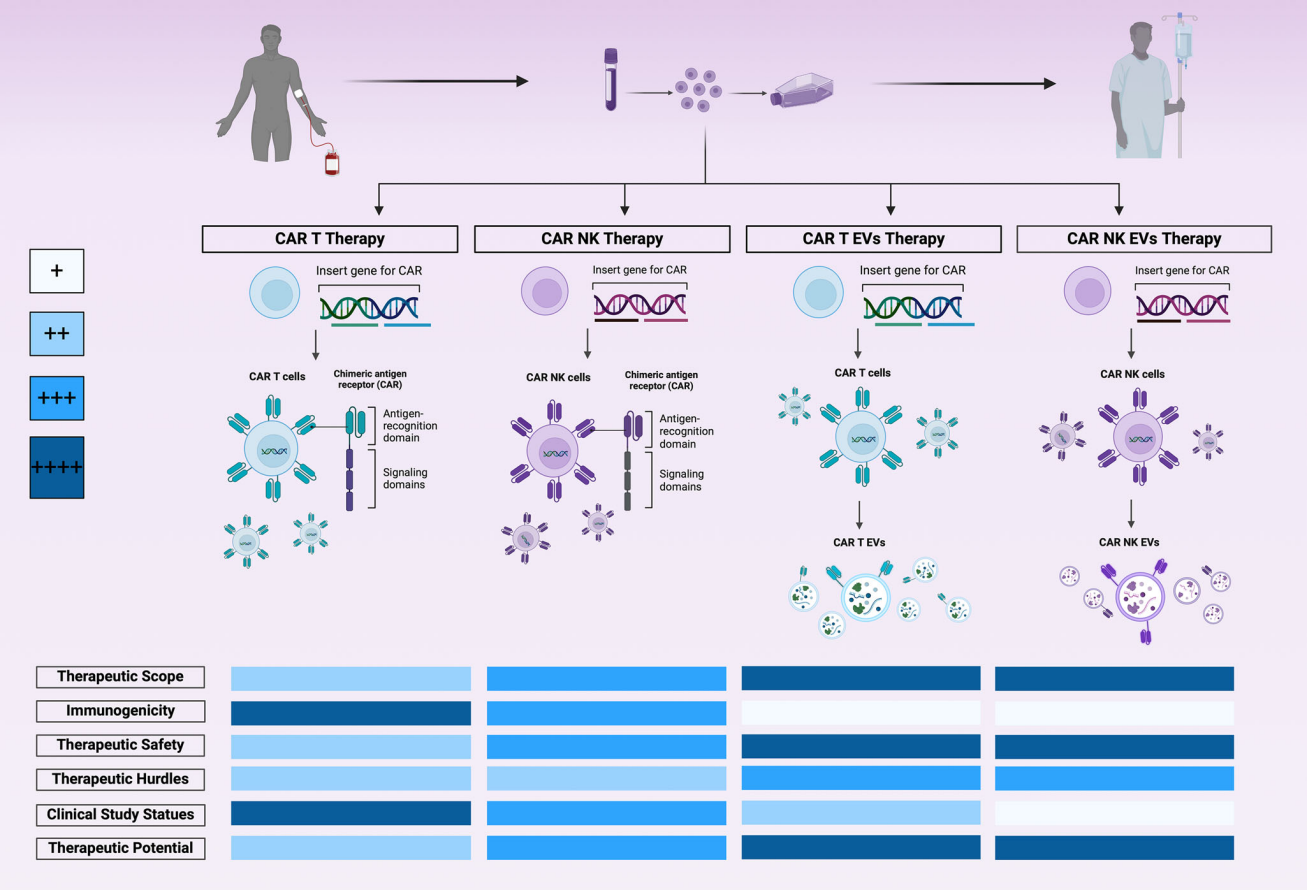
Comparing advantages and disadvantages of CAR and CAR EVs in the context of therapy.

The realm of cancer therapy is currently undergoing a dynamic transformation, characterized by the continual emergence of innovative approaches that provide hope for patients battling persistent diseases. The convergence of EVs and CAR technology presents an especially exciting frontier in cancer immunotherapy, potentially offering a multifaceted strategy to combat this complex disease. EVs have shown considerable promise as therapeutic agents in cancer treatment. Their ability to deliver tumour‐suppressing proteins, nucleic acid components, and targeted drugs has opened new avenues for precision medicine. With inherent advantages such as biocompatibility, low immunogenicity, and the capacity to cross biological barriers, EVs may become the ideal candidates for drug delivery and immunotherapy. CAR technology has revolutionized cancer immunotherapy by engineering immune cells to express CARs, enabling them to target tumour‐specific antigens. While CAR T cells have demonstrated remarkable success in treating haematological malignancies, CAR NK cells offer a distinct immunotherapeutic approach with potential advantages in safety, simplicity of manufacturing, and a broader scope for cancer treatment. The combination of CAR technology with EVs has given rise to CAR T EVs and CAR NK EVs, potentially leveraging the strengths of EVs to address challenges associated with live‐cell therapies (as can be viewed in Figure 2 and Table 1). The fusion of these two robust technologies holds immense promise in the ongoing battle against cancer. CAR T EVs and CAR NK EVs may enhance the efficacy of cancer immunotherapy by overcoming limitations associated with traditional approaches. The ability of EVs to infiltrate solid tumours, deliver precise therapeutic cargo, and modulate the immune response makes them invaluable tools in the fight against cancer. Furthermore, the potential for reduced toxicity and ‘off‐the‐shelf’ applications of CAR‐NK‐EVs makes them particularly attractive for clinical translation.

**TABLE 1:** Comparing various aspects of CAR and CAR EVs in the context of therapy.

Aspect	CAR T Cells	CAR NK Cells	CAR T EVs	CAR NK EVs
**Immunotherapeutic approach**	Adoptive cell therapy utilizing genetically engineered T cells targeting specific antigens on cancer cells	Adoptive cell therapy utilizing genetically engineered NK cells targeting specific antigens on cancer cells	Cell‐free therapy using EVs derived from CAR T cells	Cell‐free therapy using EVs derived from CAR NK cells
**Antigen recognition**	Antigen‐binding domain derived from monoclonal antibodies, providing high specificity	CAR‐mediated recognition of tumour‐specific antigens, also capable of recognizing stress ligands and downregulated MHC‐I	CAR‐T EVs express granzyme B and perforin	CAR NK EVs contains cytotoxic proteins, granzyme B, and GNLY, inducing ER stress‐mediated apoptosis
**Structure and generations (CAR)**	Antigen‐binding domain (scFv), hinge region, transmembrane domain, and intracellular signalling domains 1st to 5th generations evolving for enhanced specificity, function, and safety			
**Cancer types targeted**	Primarily used for hematologic malignancies. Treatment for solid cancers and brain tumours has been successful for specific types (HCC, DIPG)	It has shown promise in treating solid cancers and has also been effective in treating wider spectrum of hematologic malignancies.	Effective against both types of cancers (solid and hematologic). Demonstrates versatility and potential for application in a broad spectrum of cancer types.	
**Immune response**	May stimulate and harness immune response against tumour cells, with potential for long‐lasting immunologic memory	Lower toxicity potential, avoiding undesired effects in the TME, suggested to have a regulatory role in immune cell function through the delivery of effector molecules and modulation of gene expression		
**Persistence**	Long‐lasting in vivo, can lead to prolonged immune response	Generally shorter in vivo persistence	Dependent on EV formulation and targeting, typically short‐lived	
**Challenges**	CRS, on‐target/off‐tumour effects, relapses, exhaustion, tumour heterogeneity	challenges in genetic modification and expansion, and	Limited clinical data, challenges in production and standardization, potential for off‐target effects and potential safety concerns, and understanding tumour responses.	
	overcoming penetrance in solid tumours and TME suppression			
	manufacturing complexity and cost			
**Advances addressing challenges**	Advancements such as IL‐2 and safety switch receptors, and fifth‐generation CAR‐T cell therapy	Enhanced persistence and expansion techniques such as electroporation and in vivo transduction are being actively explored, while freezing methods are being optimized for "off‐the‐shelf" products, and strategies to overcome TME suppression	Optimization of production methods, engineering for enhanced targeting and therapeutic cargo loading	
	improved CAR constructs and combination therapies			
**Therapeutic utility**	Versatility to target a wide range of antigens, successful treatments for various cancer types	Promising results in haematological malignancies and solid tumours, potential for "off‐the‐shelf" applications potential for allogeneic applications	Potential for use as "off‐the‐shelf" allogeneic treatments, avoiding severe immune responses potential for targeted drug delivery and combination therapies	
**Safety profile**	Advancements are being made to reduce the side effects of treatments while maintaining their effectiveness.	Demonstrated safety in clinical trials, attributed to specific cytokine profiles limiting toxicity. Might be safer than CAR T therapy	Potentially safer than CAR T cells due to acellular nature	Might be appropriate for cancer nanomedicine due to its low immunogenicity in allogeneic settings. Might be safer than CAR T EV's therapy
			Lower toxicity potential and safer potential alternative to living CAR cell therapies	
**Clinical trials**	Ongoing and completed, Numerous ongoing clinical trials for various cancer types	Ongoing and Emerging, Increasing number of clinical trials, particularly for hematological malignancies, but less than CAR T therapy.	Emerging and Preclinical	Preclinical
			Limited clinical trials, but growing interest and research activity	
**Current research focus**	Ongoing refinement of CAR designs for improved safety and efficacy. Active research on generating sufficient CAR cells	Exploring CAR EV potential in various tumour types, compatibility with responsive and resistant tumours		

The current challenges in production, standardization, and clinical translation still stand between the potential of the field and the pharmaceutical industry. Ongoing research and development in this field are poised to unlock the full potential of these approaches, providing renewed hope to patients and clinicians alike. The convergence of EVs and CAR technology represents a significant advancement in the quest for more effective, safer, and personalized cancer treatments. Continued exploration of this dynamic frontier may well pave the way for a new era in cancer therapy, one that harnesses the power of the immune system to achieve lasting remissions and improved patient outcomes.

## AUTHOR CONTRIBUTIONS

Ofir Bar: Conceptualization (Supporting); validation(lead); writing—original draft(lead); writing—review & editing(supporting). Angel Porgador: Conceptualization(equal); writing—original draft(equal); writing—review & editing(lead). Tomer Cooks: Conceptualization(equal); supervision(lead); writing—original draft(supporting).

## CONFLICT OF INTEREST STATEMENT

The authors declare no conflicts of interest.
